# Daily Perceptions of Aging and Implications for Stress Reactivity

**DOI:** 10.1093/geroni/igab046.2105

**Published:** 2021-12-17

**Authors:** Bethany Wilton-Harding, Tim Windsor

**Affiliations:** Flinders University, Adelaide, South Australia, Australia

## Abstract

Awareness of one’s own aging has received increasing attention in the field of gerontology over the last decade. This study examines the role awareness of age-related change (AARC) may play in the association between daily stressors and well-being. Recently, individuals’ awareness of both age-related gains and age-related losses has been shown to vary on a day-to-day basis (Neupert & Bellingtier, 2017). We expected that increases in daily AARC-losses may be associated with increased emotional reactivity to daily stressors, whereas increases in AARC-gains may be associated with decreased reactivity. Data were collected in a daily diary study from a community-based sample of 152 Australian participants aged 53 to 86 (M = 69.18, SD = 5.73). Participants completed daily assessments of AARC, stressors and emotional affect (positive and negative) on their smartphones for 10 consecutive days. Analysis of within-person coupling using multilevel models indicated that daily increases in AARC-losses were associated with increased reactivity to daily stressors (represented by high negative affect and low positive affect). On the other hand, daily increases in AARC-gains were associated with decreased reactivity to daily stressors (represented by low negative affect). Results indicate that even short-term fluctuations in perceptions of aging may be an important factor to consider when investigating associations between daily stressors and well-being in older adulthood. Specifically, greater daily AARC-losses may contribute to lower emotional well-being, whereas an appreciation of positive age-related changes (AARC-gains) may play a role in mitigating emotional reactivity to daily stress experiences in older adulthood.

